# Static Load Characteristics of Hydrostatic Journal Bearings: Measurements and Predictions

**DOI:** 10.3390/s22197466

**Published:** 2022-10-01

**Authors:** Howon Yi, Hyunsung Jung, Kyuman Kim, Keun Ryu

**Affiliations:** 1 Department of Mechanical Design Engineering, Hanyang University, Seoul 04763, Korea; 2 Department of Mechanical Engineering, BK21 FOUR ERICA-ACE Center, Hanyang University, Ansan 15588, Gyeonggi-do, Korea; 3 Department of Mechanical Engineering, Hanyang University, Ansan 15588, Gyeonggi-do, Korea

**Keywords:** hydrostatic bearings, journal bearings, static load characteristics, measurements, predictions

## Abstract

Hydrostatic bearings for liquid rocket engine turbopumps provide distinctive advantages, including high load capacity even with low viscosity cryogenic fluid and extending life span by minimizing friction and wear between rotor and bearing surfaces. Application of hydrostatic bearings into turbopumps demands a reliable test database with well-quantified operating parameters and experimentally validated accurate performance predictive tools. The present paper shows the comprehensive experimental data and validation of predicted static load characteristics of hydrostatic journal bearings lubricated with air, water, and liquid nitrogen. Extensive experiments for static load characteristics of hydrostatic bearings are conducted using a turbopump-rotor-bearing system simulator while increasing supply pressure (*P_s_*) into the test bearings. The test results demonstrate notable effects of the test fluids and their temperatures, as well as *P_s_*, on the bearing performance. In general, the measured bearing flow rate, rotor displacement, and stiffness of the test bearings steadily increase with *P_s_*. The static load bearing characteristics predictions considering flow turbulence and compressibility matched well with the experimental results. The work with independent test data and engineering computational programs will further the implementation of hydrostatic bearings in high-performance turbopump shaft systems with improved efficiency and enhanced reusability of liquid rocket engine sub-systems.

## 1. Introduction

Hydrostatic bearings in high-speed rotating machinery enable low wear, insignificant friction, and accurate shaft positioning. In particular, hydrostatic bearings offer large load capacity and stiffness, even when lubricated with a low-viscosity fluid. These advantages allow hydrostatic bearings to be successfully applied in cryogenic applications, such as turbopumps for liquid rocket engines [1,2,3,4,5]. Moreover, hydrostatic bearings are a promising technology for reusable liquid rocket engines [6,7,8]. 

The successful deployment of hydrostatic bearings into high-performance cryogenic applications requires comprehensive experimental measurements and reliable characteristics predictions [9,10,11,12]. Note that it is common that performance measurements of cryogenic bearings for liquid rocket engine turbopumps are conducted using warm or hot water as a test fluid. Kurtin et al. [4] conducted measurements and predictions of static load characteristics (such as shaft-bearing relative position, torque, recess pressure, and flow rate) of a water-lubricated hydrostatic journal bearing. The operating speed ranged from 10 krpm to 25 krpm. The water supply pressures into the bearing were 6.89 MPa, 5.52 MPa, and 4.14 MPa. The test hydrostatic bearing was a five-recess, orifice-compensated hydrostatic bearing with 76.2 mm in bearing diameter and 76.2 mm in bearing axial length. The purified water at 54 °C was used as a test fluid to achieve comparatively high Reynolds numbers in the test bearing without using a cryogenic fluid. The bulk-flow bearing model predicted bearing characteristics. Franchek and Childs [13] measured bearing flow rate and dynamic force coefficients of four hydrostatic bearings with different recess configurations (a square-recess bearing, a smooth-land bearing, a radial-orifice bearing, and a circular-recess bearing). The measurements were conducted at a rotor speed of 24.6 krpm for supplying pressure of 4 MPa and 7 MPa with purified water at 55 °C. The diameter of each test bearing and the bearing *L*/*D* (i.e., length/diameter) ratio were 76.2 mm and 1, respectively. The test results showed that the flow rate slightly decreased with rotor speed. The bearing stiffnesses increased with rotor speed and are invariant with the bearing eccentricity ratio. Testing and demonstration of hydrostatic bearings using a cryogenic fluid are also required to handle more challenging, extreme, and realistic operating conditions for turbopump applications. For example, Oike et al. [6] conducted experiments on static and dynamic load characteristics (such as recess pressure ratio, flow coefficient, and stiffness) of hydrostatic bearings lubricated with liquid nitrogen. The test hydrostatic bearing, with young leaf mark recesses, has a diameter of 60 mm, a length of 25 mm, and a radial clearance of 0.055 mm. The experimental demonstration showed that the coated surface damage of the test hydrostatic bearing during extensive rotating tests does not significantly affect the overall bearing performance.

Hydrostatic bearing performance largely relies on turbulence and inertia effects in a cryogenic operating condition. San Andrés [14,15,16] introduced a turbulent inertia bulk flow model for prediction of isothermal characteristics of hydrostatic bearings for cryogenic applications. The results showed that the fluid inertia effect in high-speed cryogenic bearings reduced bearing flow rates and enhanced hydrodynamic effects. Later, San Andrés et al. [17] introduced a bulk-flow thermo-hydrodynamic model to predict the static and dynamic load characteristics of orifice-compensated hydrostatic bearings for cryogenic applications. The predicted results showed a good correlation with experimental data from a water-lubricated hydrostatic bearing. The work demonstrated the accuracy of the adiabatic flow thermos-hydrodynamic analysis for cryogenic fluid film bearings. Yoshikawa et al. [18] calculated the stiffness and damping coefficients of cryogenic hydrostatic bearings for liquid hydrogen turbopumps. Bearing dynamic load characteristics were predicted using the Reynolds equation considering a turbulent effect. The predicted results showed that the effects of rotor speed, bearing eccentricity, and bearing recess design parameters on the bearing performance. 

Presently, exhaustive measurements are conducted for bearing flow rate, bearing orifice discharge coefficient, bearing torque, rotor centerline motion, and bearing stiffness with increasing supply pressure (*P_s_*) into the test bearings at a non-rotating condition prior to extensive rotordynamic tests of the hydrostatic journal bearing supported rotor system. The non-rotating operating condition is intended to eliminate the contribution of hydrodynamic pressure within the test bearings on the bearing performance and characteristics. That is, the current work only shows the bearing static load performance due to external pressurization of a test fluid into the test bearings. Air (bearing inlet temperature 25 °C, which is a controlled room temperature), water (bearing inlet temperature 6 °C, 25 °C, 48 °C, and 70 °C), and liquid nitrogen (bearing inlet temperature −197 °C) are used as test fluids to demonstrate the effects of lubricant properties and conditions on the bearing performance. The feed pressure condition of each test fluid is manipulated to identify its effect on the static load characteristics of the test bearings. In addition, the measurements are compared with the predictions.

The current work details a direct comparison of static load characteristics of hydrostatic bearings tested with compressible (i.e., air), incompressible (i.e., water), and cryogenic (i.e., liquid nitrogen) fluids using the same test bearings in a rotordynamic test simulator system in non-rotating conditions to distinguish the effect of hydrostatic pressure into the bearings. In addition, the work demonstrates the notable effects of the physical properties and supply conditions of the test fluids on the performance of hydrostatic bearings. Note that no archival literature shows experimental efforts that directly compare the static load performance of hydrostatic bearings tested with compressible, incompressible, and cryogenic fluids.

## 2. Experimental Facility

**Figure 1:**
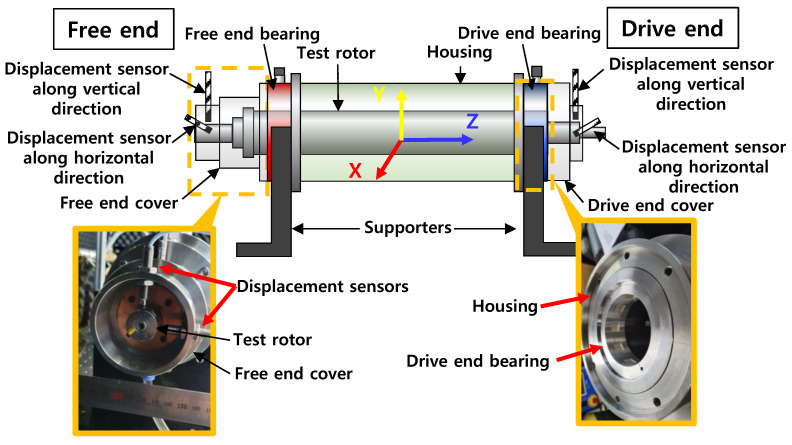
Schematic view (not to scale) and photographs of current test rig.

**Figure 2:**
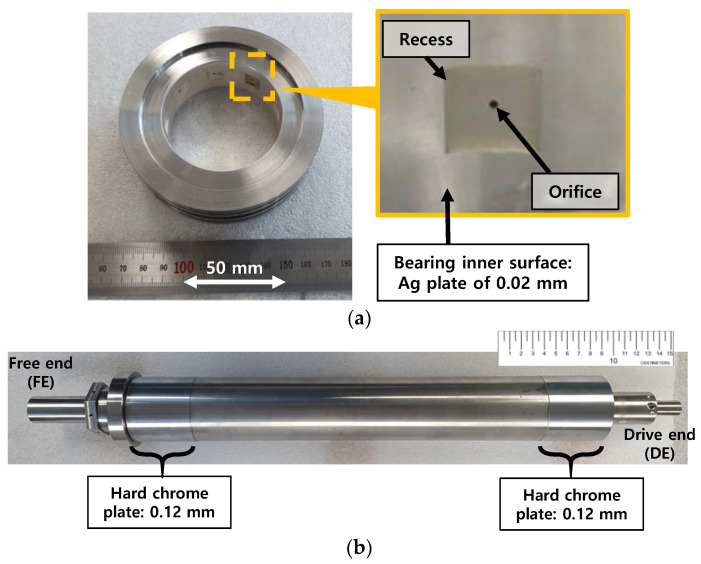
Photographs of test hydrostatic baring and test rotor. (**a**) Test bearing. (**b**) Test rotor.

Figure 1 depicts a schematic view of the hydrostatic bearing supported rotordynamic test rig simulating a cryogenic turbopump rotor-bearing system. The test rig consists of a rigid steel bearing housing, two housing supporters, two side covers, two test hydrostatic bearings, and the test rotor. Figure 2 depicts photographs of a test hydrostatic bearing and a test rotor. Table 1 lists the main dimensions and physical properties of the test rotor and the test bearings. The test rotor, 10.40 kg in mass, is a SUS 630 shaft 590 mm in length and ~59.9 mm in outer diameter at the bearing locations. The transverse moment of inertia (*I_t_*) and polar moment of inertia (*I_p_*) of the test rotor are 2.12 × 10^−1^ kg·m^2^ and 4.61 × 10^−3^ kg·m^2^, respectively. Hard chrome plating (0.12 mm in thickness) is applied to the rotor outer surfaces at the bearing location. The center of mass of the test rotor is 293 mm from the rotor free end. The fraction of rotor weight acting on the free end and the drive end bearings equals ~55 N and ~47 N, respectively. The test bearings, made of SUS 630, are orifice-compensated-type hydrostatic bearings with nine square recesses (0.823 mm in depth). The inner diameter and axial length of both test bearings are ~60 mm and 25 mm, respectively. The bearing inner surfaces are coated with Ag (i.e., ~0.02 mm thickness silver plating). The axial and the circumferential lengths of the recesses fabricated on the bearing inner surfaces are both ~9.86 mm. The outer diameter of the test bearings has circumferential grooves as a fluid path for pressurized lubricant. The bearing outer surfaces also have circumferential grooves for insertion of O-rings. Note that the current bearing recess and orifice configurations follow the design approach and dimensions detailed in Refs. [19,20,21] for rocket engine turbopump applications. A separate and independent parametric study of the bearing recess and orifice dimensions (not shown here for brevity) confirms that the current bearings are capable of supporting a shaft system in rocket engine cryogenic turbopumps. Rotor displacements are recorded using two pairs of displacement sensors located at the free end rotor and drive end rotor. The displacement sensors are orthogonally affixed on each side cover.

**Figure 3:**
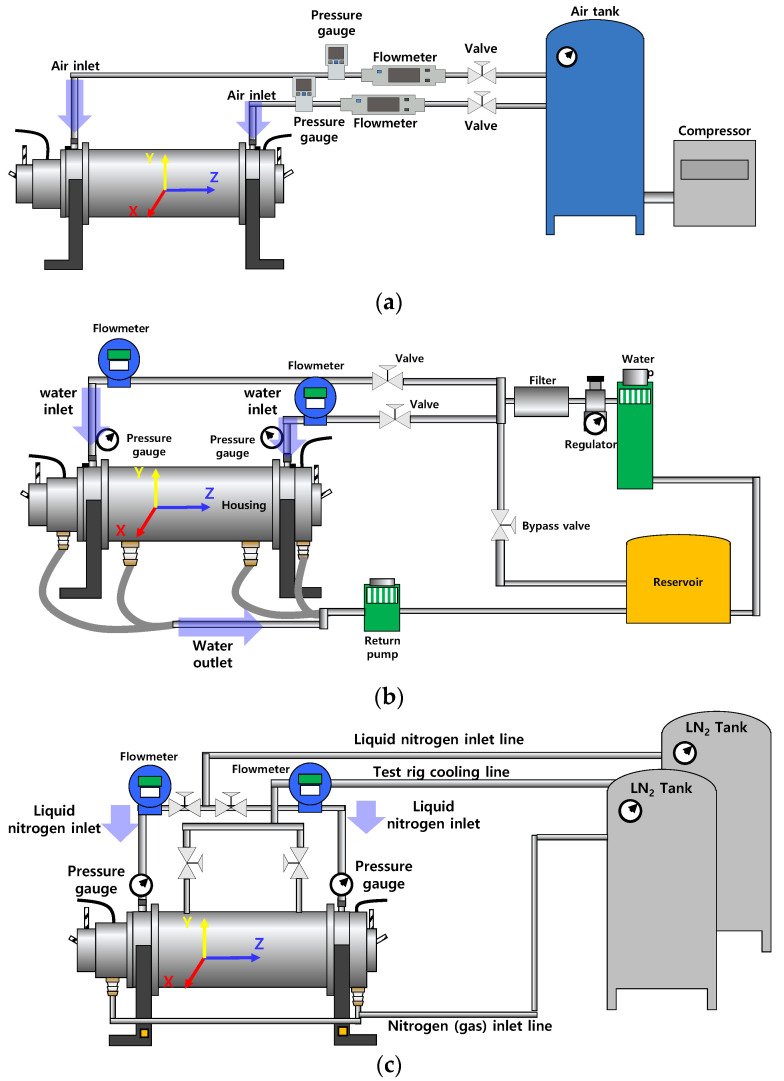
Schematic view (not to scale) of test fluid supply systems. (**a**) System configuration for air-lubricated bearing tests. (**b**) System configuration for water-lubricated bearing tests. (**c**) System configuration for liquid-nitrogen-lubricated bearing tests.

Figure 3a–c depict schematic views of the test fluid supply systems (air for Figure 3a, water for Figure 3b, and liquid nitrogen for Figure 3c) into the test bearings. The test fluid for bearing lubrication enters into each test bearing through steel pipes installed at the housing. The pressure gauges and the flowmeters measure and record the pressure and flow rate of the test fluids fed into the test bearings. For air-lubricated bearing tests (Figure 3a), the air flow meters measure the flow rate up to 200 L/min with ±1.5% of full-scale accuracy. The air pressure gauges measure the pressure up to 10 bar(g) with ±2% of full-scale accuracy. For water-lubricated bearing tests (Figure 3b), the water flow meters measure the flow rate up to 20 L/min with ±0.5% of full-scale accuracy. The water pressure gauges measure the pressure up to 15 bar(g) with ±1.5% of full-scale accuracy. For liquid-nitrogen-lubricated bearing tests (Figure 3c), the cryogenic liquid nitrogen flow meters measure the flow rate up to 60 L/min with ±0.5% of full-scale accuracy. The cryogenic pressure gauges measure the pressure up to 35 bar(g) with ±1.5% of full-scale accuracy.

## 3. Test Cases and Experimental Methods

Table 2 lists the present test cases. Test cases #1, #2, and #3 use pressurized air, water, and liquid nitrogen into the test bearings, respectively, as a test fluid. For the air-lubricated bearing tests (i.e., test case #1), air at 25 °C (controlled bearing inlet temperature) as a test fluid is fed into the bearings. *P_s_* increases from 0.1 bar(g) to 1.6 bar(g) in 0.1 bar(g) increments. The measured parameters are *P_s_*, flow rate, rotor displacement, and torque. From the measured data, bearing orifice discharge coefficients and bearing stiffness coefficients along horizontal direction are estimated. Note that when air (i.e., test case #1) or liquid nitrogen (i.e., test case #3) is fed into the test bearings, pneumatic hammer instability occurs even with *P_s_* =1 bar(g). For test cases #1 and #3, pneumatic hammer instability becomes more distinctive as *P_S_* increases. Therefore, to prevent damage to the test bearings and the test rotor, measurements are conducted up to *P_s_* =1.6 bar(g) for test case #1 and up to *P_s_* = 4 bar(g) for test case #3. Note, for test cases #1 and #2, flow rate and *P_s_* are measured for both the bearing-only (i.e., without rotor in the test bearings) and the rotor-bearing conditions (i.e., rotor inserted in the test bearings). However, in test case #3, flow rate and *P_s_* are measured for the bearing-only condition. Note that the test fluid temperature is monitored at the inlet locations of the test bearing.

Orifice discharge coefficients of the test bearings are estimated using the recorded flow rate and *P_s_*. The flow rate through an orifice is calculated using

(1) $$Q=C_{d}A_{o}\sqrt{\frac{2\left(P_{s}-P_{r}\right)}{\rho }}$$

where $C_{d}$ is an orifice discharge coefficient (the ratio of the flow rate through the orifice to the theoretical flow rate), $A_{o}$ is an orifice area ($A_{o\;}$=$\;\frac{\pi d_{o}}{4}$), $P_{s}$ is supply pressure, $P_{r}$ is recess pressure, and ρ is density. The flow equation for compressible fluid is expressed as

(2a) $$Q=C_{d}A_{o}\frac{P_{s}}{\rho \left(P_{r}\right){\left(ℜT_{s}\right)}^{\frac{1}{2}}}\Phi g$$

(2b) $$g={\left[1+{\left(\frac{d_{orifice}}{4\left(c+h_{recess}\right)}\right)}^{2}\right]}^{-\frac{1}{2}}$$

(2c) $$\Phi ={\left(\frac{2\kappa }{\kappa -1}\right)}^{\frac{1}{2}}{\left(\frac{P_{r}}{P_{S}}\right)}^{\frac{1}{\kappa }}\times {\left[1-{\left(\frac{P_{r}}{P_{S}}\right)}^{\frac{\left(\kappa -1\right)}{\kappa }}\right]}^{\frac{1}{2}},\;\mathrm{if}\;\frac{P_{r}}{P_{s}}>0.528$$

(2d) $$\Phi ={\left(\frac{2\kappa }{\kappa +1}\right)}^{1/2}\times {\left(\frac{2}{\kappa +1}\right)}^{1/\left(\kappa -1\right)},\;\mathrm{if}\;\frac{P_{r}}{P_{s}}\leq 0.528$$

where ℜ is a gas constant, $T_{s}$ is temperature of a supply fluid, $d_{orifice}$ is a diameter of an orifice, g is a flow function, and κ is a specific heat ratio of air.

**Figure 4:**
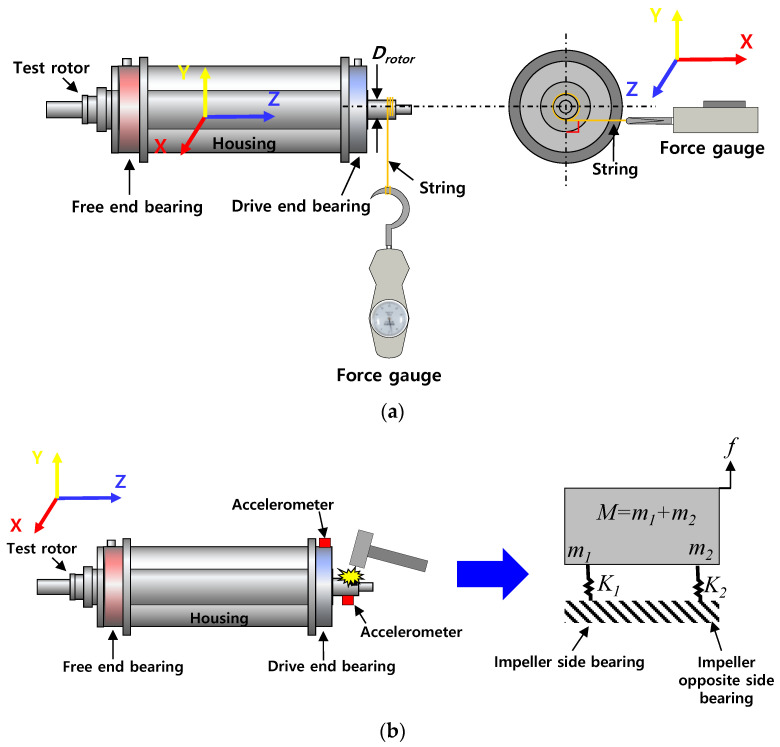
Schematic view (not to scale) of (**a**) torque measurement and (**b**) rap test for stiffness estimation.

Figure 4a,b show schematic views (not to scale) of the bearing torque measurement and the rap test for bearing stiffness estimation. Figure 4b also shows a physical model of the rotor-bearing system. As shown in Figure 4a, the bearing torque is measured to determine the lowest *P_S_* for complete lift-off (i.e., no physical contact) of the test rotor from the bearing inner surfaces. The force is measured while pulling the sting connected between the test rotor and the force gauge. The bearing torque is measured by multiplying the force applied to the rotor by the radius of the rotor at the bearing locations. Each torque measurement result uses the average value of the data measured thrice. A series of rap tests is conducted while supplying a test fluid to the test bearings to estimate bearing stiffnesses. The bearing stiffness (*K*) is identified by the measured acceleration response obtained from the rap test. The accelerometer is attached to one end of the test rotor. Note that $K=\omega_{n}^{2}M$, where *K* is a bearing stiffness, $\omega_{n}$ is a measured natural frequency, and *M* is the rotor weight. In Figure 4b, *m_1_* and *m_2_* represent the static load acting on the free end bearing and the drive end bearing, respectively. In addition, *K_1_* and *K_2_* indicate the stiffness of each test bearing. Presently, all measurements are conducted thrice under static steady-state conditions and the average value of three results is shown used for the test result.

## 4. Experimental Results

### 4.1. Flow Rate and Orifice Discharge Coefficient of Test Bearings

**Figure 5:**
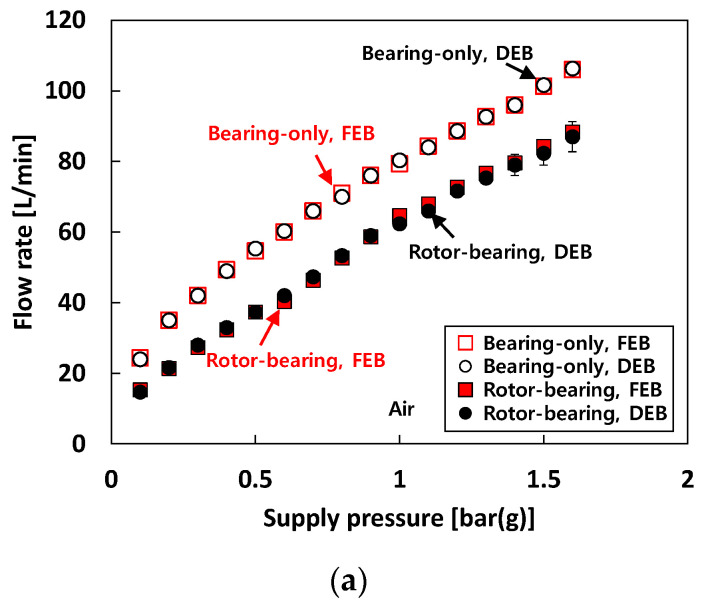

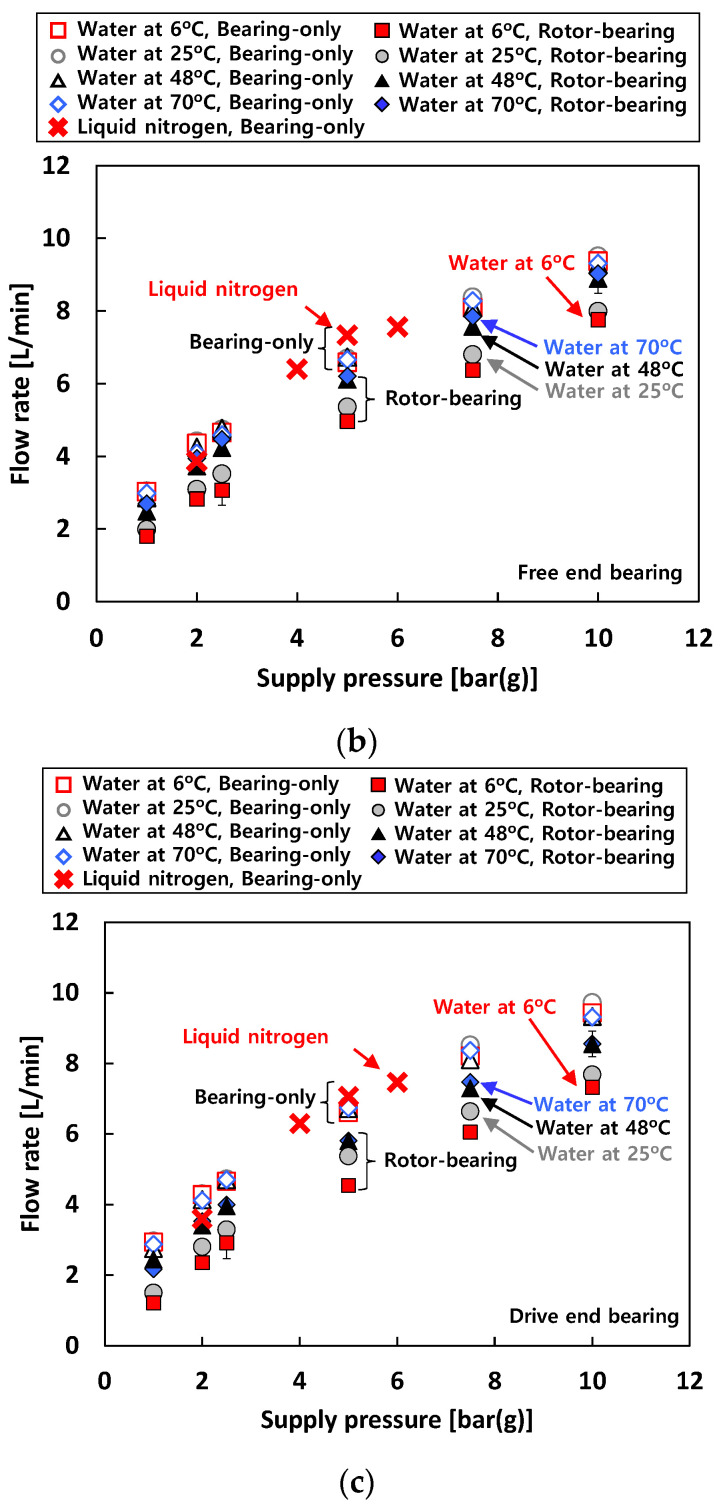
Test cases #1 through #3: recorded flow rate versus supply pressure. (**a**) Test case #1. DEB: drive end bearing. FEB: free end bearing. (**b**) Test cases #2 and #3. Free end bearing. (**c**) Test cases #2 and #3. Drive end bearing.

Figure 5 presents the recorded flow rate versus *P_s_* into the test bearings for each test fluid. The bearing flow rates are measured for both the bearing-only (i.e., without rotor in the test bearings) and the rotor-bearing (i.e., rotor inserted in the test bearings) conditions. Recall that the loads acting on the free end bearing and drive end bearing are 55 N and 47 N, respectively. When 25 °C air is used as a test fluid (i.e., test case #1), the flow rate increases linearly as *P_s_* increases for both the bearing-only and rotor-bearing conditions. Obviously, at the same *P_s_*, the measured bearing flow rates at the bearing-only condition are larger than those measured at the rotor-bearing condition. The flow rate difference between the rotor-bearing and the bearing-only conditions is nearly constant (~16 L/min) with increasing *P_S_*. Note that there is no notable difference in flow rate between the free end bearing and the drive end bearing. Recall that pneumatic hammer instability occurs from *P_s_* = 1 bar(g) and the vibration amplitude caused by pneumatic hammer instability increases with *P_s_*. 

For test case #2 (i.e., when water is used as a test fluid), the flow rates increase linearly with *P_s_* for both the bearing-only and rotor-bearing conditions. Note that for test case #2, at the same *P_s_*, the measured bearing flow rates increase with temperature of the test fluid for the rotor-bearing condition due to the changes in bearing eccentricity. On the other hand, there is no noticeable difference in flow rate with increasing temperature of the test fluid for the bearing-only condition. 

Interestingly, the recorded flow rates for test case #3 (i.e., when liquid nitrogen is used as a test fluid) are quite similar with the flow rates when 70 °C water is used as a test fluid under the bearing-only condition. Note that, as shown in Equation (1), the bearing flow rate mostly relies on the fluid property and the orifice geometry which significantly vary with temperature. Therefore, a combined contribution of the differences in the test fluid density and the orifice diameter due to the ~270 °C temperature change (between 70 °C water and liquid nitrogen) makes the flow rates for test case #3 similar to those for test case #2 with 70 °C water.

The test results show that at the same *P_s_*, the bearing flow rates for the air-lubricated condition (i.e., test case #1) are larger than those for the water-lubricated (i.e., test case #2) and liquid-nitrogen-lubricated (i.e., test case #3) conditions. The recorded flow rates of the free end bearing and the drive end bearing under the bearing-only condition show similar values. Note that for the rotor-bearing condition under the same *P_s_*, the flow rates of the free end bearing are always slightly higher than those of the drive end bearing.

**Figure 6:**
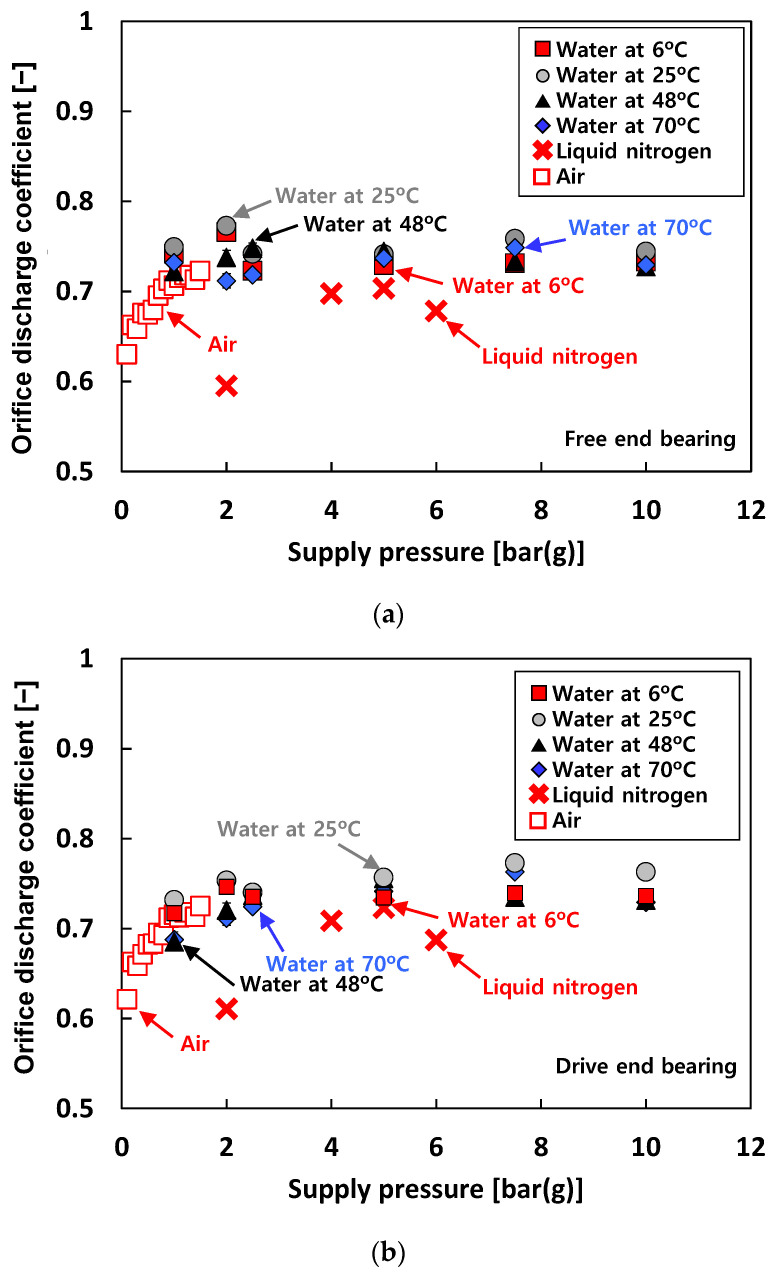
Test cases #1 through #3: estimated orifice discharge coefficient (*C_d_*) versus supply pressure (*P_s_*). (**a**) Free end bearing. (**b**) Drive end bearing. Note: The lowest value of the *Y*-axis is not 0 but 0.5.

Figure 6 depicts the estimated orifice discharge coefficients (*C_d_*) versus *P_s_*. The orifice discharge coefficients are estimated using Equation (1) for water and liquid nitrogen and Equation (2a) for air. Note that the physical properties of air, water, and liquid nitrogen are taken from Refs. [22,23,24,25,26]. When air is used as a test fluid (i.e., test case #1), *C_d_* gradually increases while *P_s_* increases from 0.1 bar(g) to 1 bar(g), then shows an almost constant value of ~0.72 above 1 bar(g). When water is used as a test fluid (i.e., test case #2), *C_d_* shows a nearly invariant value of ~0.74. In addition, for test case #2, the difference in *C_d_* with increasing temperature of the test fluid is not notable. For test case #3, *C_d_* ranges from 0.6 to 0.7. The differences in *C_d_* for the free end bearing and the drive end bearing are not noticeable.

### 4.2. Rotor Centerline Motions and Bearing Eccentricity Ratio

**Figure 7:**
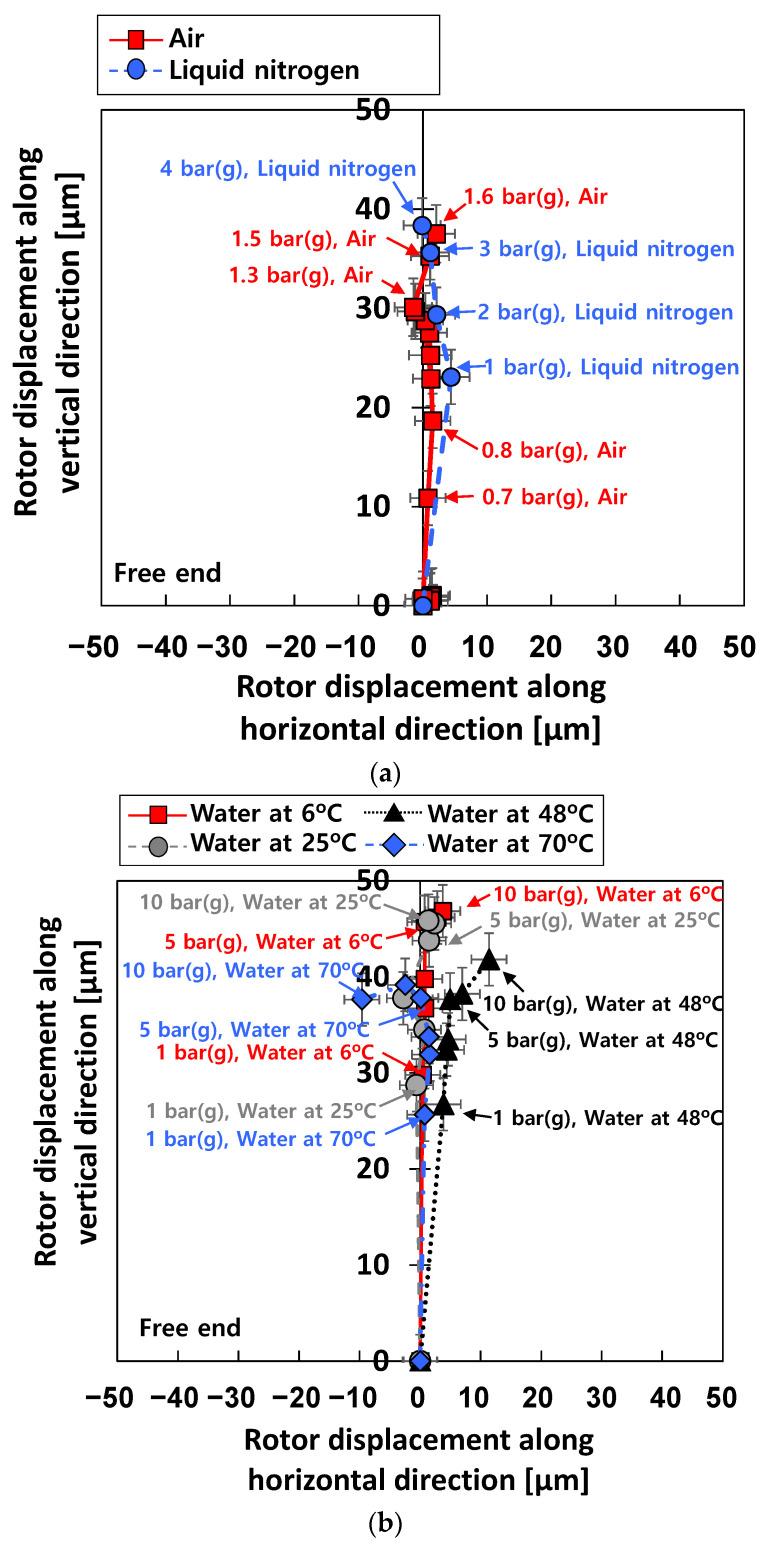
Test cases #1 through #3: measured rotor centerline motions for increasing *P_s_*. Free end bearing. Static load on the bearing: ~55 N. (**a**) Test cases #1 and #3. (**b**) Test case #2.

Figure 7 depicts the measured centerline travel (i.e., the static displacement of the rotor centerline) of the test rotor at the free end for increasing *P_s_*. The initial position of the test rotor (i.e., the coordinates (0, 0) in Figure 7) denotes the rotor position within the test bearing when *P_s_* = 0 bar(g). The centerline of the test rotor increases along the vertical plane as *P_s_* increases. For test case #1 (i.e., tests with air), the test rotor is lifted off from the bottom of the bearing inner surface to ~25% of the (room temperature assembly) bearing diametrical clearance for *P_s_* = 1 bar(g). For test case #3 (i.e., tests with liquid nitrogen), the test rotor is lifted off from the bottom of the bearing inner surface to ~23% of the (room temperature assembly) bearing diametrical clearance for *P_s_* = 1 bar(g). For test case #2 (i.e., tests with water) with *P_s_* = 1 bar(g), the test rotor is lifted off from the bottom of the bearing inner surface to ~31%, ~28%, ~26%, and ~25% of the (room temperature assembly) bearing diametrical clearance for at 6 °C water, 25 °C water, 48 °C water, and 70 °C water, respectively.

**Figure 8:**
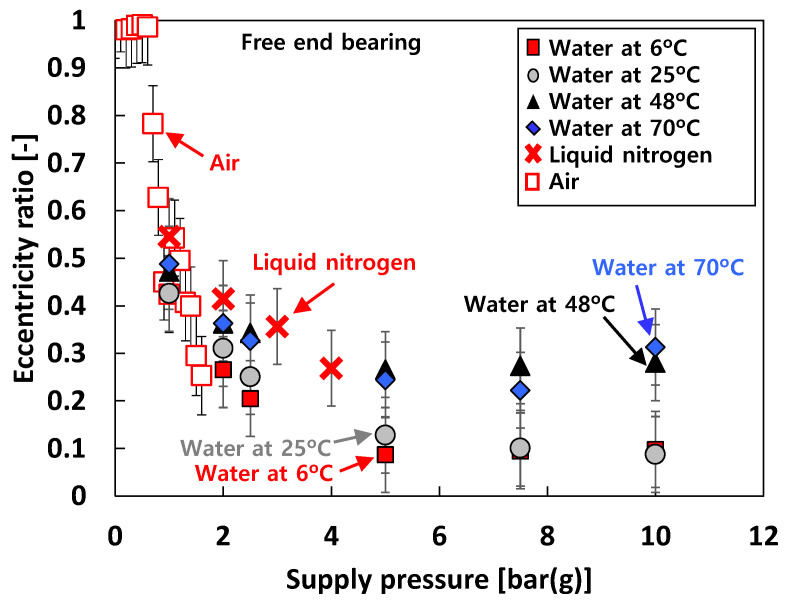
Test cases #1 through #3: measured bearing eccentricity ratio versus supply pressure. Free end bearing. Static load on the bearing: ~55 N.

Figure 8 depicts the measured eccentricity ratio versus *P_s_* for the free end bearing. The bearing eccentricity ratios rapidly decrease as *P_s_* increases when *P_s_* ranges from *P_s_* = 0 bar(g) to *P_s_* = ~5 bar(g), then becomes nearly invariant to *P_s_* when *P_s_* > 5 bar(g). For test case #2 (i.e., tests with water), at the same *P_s_*, the eccentricity ratios decrease as the test fluid temperature increases. Note that the eccentricity ratios for test case #3 (i.e., tests with liquid nitrogen) are larger than test case #2 (i.e., tests with water).

### 4.3. Bearing Torque

**Figure 9:**
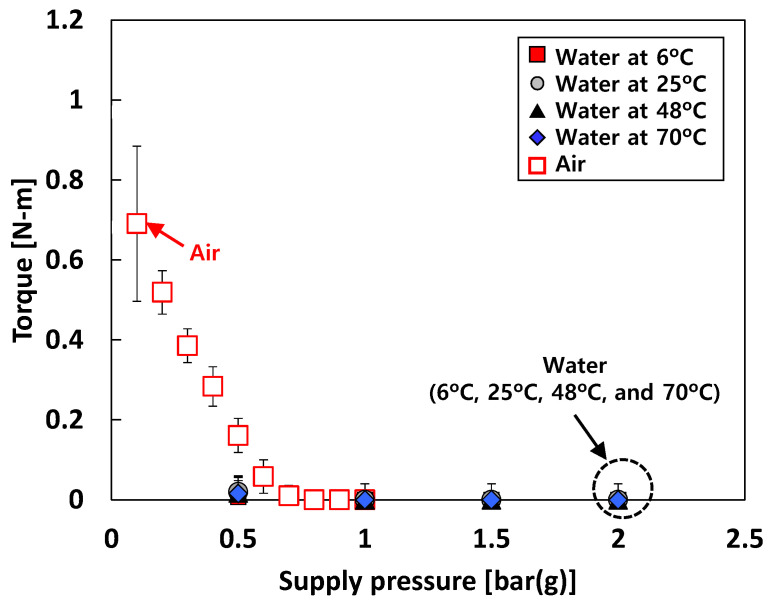
Test cases #1 and #2: measured bearing torque versus supply pressure.

Figure 9 depicts the measured bearing torque versus *P_s_* for test cases #1 and #2. Note that (nearly) zero bearing torque represents complete separation (i.e., lift-off) of the rotor surface from the bearing surfaces due to fluid external pressurization. For test case #1 (i.e., tests with air), the measured bearing torques at *P_s_* > 0.8 bar(g) are ~0 N-m. For test case #2 (i.e., tests with water), the measured bearing torques at *P_s_* > 1 bar(g) are ~0 N-m. Interestingly, for test case #1, when *P_s_* < 0.6 bar(g), the bearing torque linearly decreases with *P_s_*.

Bearing torque measurements were not conducted for test case #3 (i.e., tests with liquid nitrogen) at the time of the current experimental study due to difficulty in the test setup in a cryogenic condition. This limitation can be resolved in future experimentation.

### 4.4. Bearing Stiffness

**Figure 10:**
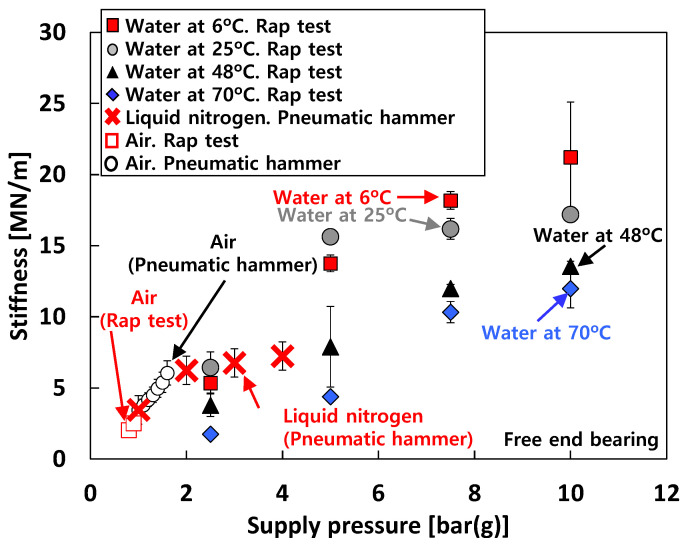
Test cases #1 through #3: stiffness versus supply pressure. Free end bearing.

Figure 10 depicts the measured stiffnesses (*K*) of the test bearings from the rap tests (recall Figure 4b) for increasing *P_s_*. The figure also includes the identified bearing stiffnesses from the excited frequencies due to pneumatic hammer instability for test cases #1 and #3. For test case #1 (i.e., tests with air), pneumatic hammer instability occurs when *P_s_* > 1 bar(g). Therefore, when *P_s_* < 1 bar(g), the natural frequencies are identified from the rap test, while those are identified from the frequencies excited by pneumatic hammer instability when *P_s_* > 1 bar(g). The bearing stiffnesses for test case #2 (i.e., tests with water) are estimated by the rap test. For test case #3 (i.e., tests with liquid nitrogen), when *P_s_* < 4 bar(g), it is important to note that due to insufficient thermal insulation around the test bearings in the bearing housing and low *P_s_* for external pressurization into the bearings, the test fluid (i.e., liquid nitrogen) experiences a phase transition from all-liquid to two-phase (liquid–gas) flow in the thin bearing films. This two-phase fluid condition in cryogenic bearings for turbopump applications is not uncommon, see Refs. [27,28]. The test results clearly show significant effects of test fluids and bearing inlet fluid temperature (*T_s_*) on the measured bearing stiffnesses.

For test case #1 (i.e., tests with air), the measured stiffnesses linearly increase with *P_s_*. For test case #1, *K* for *P_s_* = 1.6 bar(g) ≈ ~2 × *K* for *P_s_* = 0.8 bar(g). For test case #3 (i.e., tests with liquid nitrogen), the *K* rapidly increases with *P_s_* when *P_s_* < 2 bar(g) while the *K* slightly increases with *P_s_* when 2 bar(g) < *P_s_* < 4 bar(g). That is, for test case #3, *K* for *P_s_* = 2 bar(g) ≈ ~2 × *K* for *P_s_* = 1 bar(g) while *K* for *P_s_* = 4 bar(g) ≈ ~1.1 × *K* for *P_s_* = 2 bar(g). For test case #2 (i.e., tests with water), the *K* gradually increases with *P_s_* and tends to increase as the temperature of the test fluid decreases. For tests with 70 °C water, *K* for *P_s_* = 5 bar(g) ≈ ~2.5 × *K* for *P_s_* = 2.5 bar(g) and *K* for *P_s_* = 10 bar(g) ≈ ~2.75 × *K* for *P_s_* = 5 bar(g). For tests with 6 °C water, *K* for *P_s_* = 5 bar(g) ≈ ~2.6 × *K* for *P_s_* = 2.5 bar(g) and *K* for *P_s_* = 10 bar(g) ≈ ~1.5 × *K* for *P_s_* = 5 bar(g). Note that, *K* for *T_s_* = 6 °C ≈ ~3.2 × *K* for *T_s_* = 70 °C when *P_s_* = 5 bar(g) and *K* for *T_s_* = 6 °C ≈ ~1.8 × *K* for *T_s_* = 70 °C when *P_s_* = 10 bar(g). 

## 5. Predictions and Comparison to Measurements

The present study employs the Reynolds equation considering the turbulent effect and compressibility of fluid film in the bearings to predict the bearing performance. Note, see Ref. [29] for details on the current physical model and numerical method for hydrostatic bearings.

**Figure 11:**
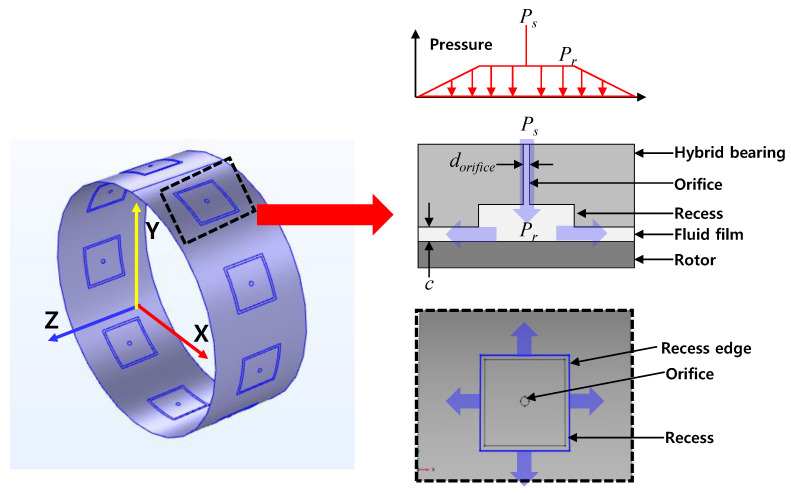
Hydrostatic bearing predictive model and flow path at recess.

Figure 11 shows an orifice-compensated hydrostatic bearing finite element model with nine recesses. The nine recesses are uniformly distributed along the circumferential direction on the inner surface of the bearing. The pressurized fluid (i.e., lubricant) is fed into each recess through the orifice with *P_s_* and then flows out of the recesses. The steady-state Reynolds equation for an isothermal and isoviscous fluid film is written as

(3a) $$\frac{\partial }{\partial x}\left(\frac{1}{G_{x}}\frac{\rho h^{3}}{\mu }\frac{\partial P}{\partial x}\right)+\frac{\partial }{\partial z}\left(\frac{1}{G_{z}}\frac{\rho h^{3}}{\mu }\frac{\partial P}{\partial z}\right)=\frac{U}{2}\frac{\partial \left(\rho h\right)}{\partial x}+\frac{\partial \left(\rho h\right)}{\partial t}$$

(3b) $$G_{x}=12+0.136Re^{0.90}$$

(3c) $$G_{z}=12+0.0043Re^{0.96}$$

where P is the pressure of the fluid film, U is the rotor surface speed, $G_{x}$ and $G_{z}$ are turbulence parameters, ρ and μ are density and viscosity of the lubrication fluid. Note that the turbulence parameters are 12 for laminar flow. It is important to note that the present bearing predictive model assumes a single-phase condition in a bearing film.

The inlet flow rate of each recess from the orifice is calculated using Equation (1) for incompressible fluid and Equation (2a) for compressible fluid. Note that the orifice discharge coefficient used in Equations (1) and (2a) are determined from the test data.

The boundary conditions of the hydrostatic bearing model are as follows. The film pressure of the edge of the bearing is the ambient pressure. The total inlet flow supplied through the orifice equals the total outlet flow exiting the recess. Flow continuity through the bearing recess using the flow equations defines the pressure in the recesses (*P_r_*), recall Equations (1) and (2a–d). That is, *P_s_*, *Q_r_*, and *C_d_* mainly determine *P_r_*. See Refs. [14,30] for further details on the fundamental model of multi-recess hydrostatic bearings which is employed in the present work.

**Figure 12:**
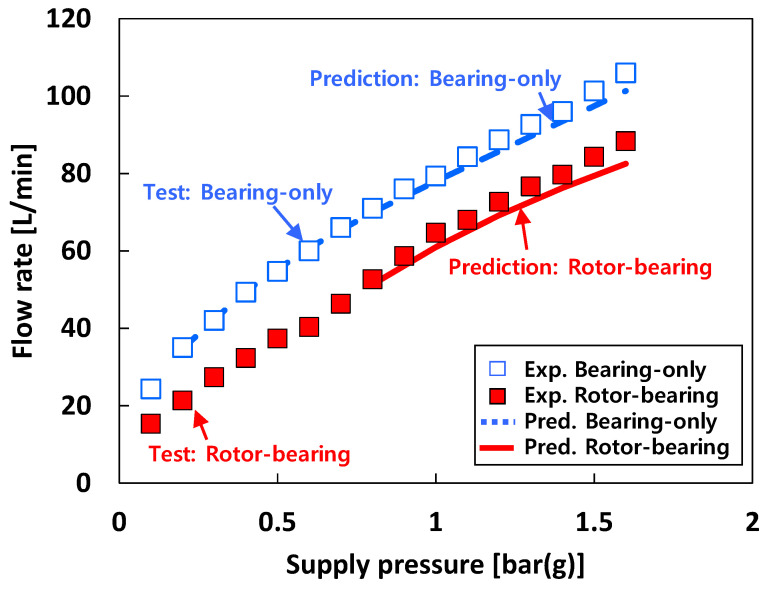
Predictions versus measurements. Test case #1: bearing flow rate of free end bearing.

For test case #1 (i.e., tests with air), Figure 12 compares the predicted and the measured flow rate of the free end bearing. Both measurements and predictions show that the flow rates for the bearing-only condition (i.e., without rotor in the test bearings) are higher than those for the rotor-bearing condition (i.e., rotor inserted in the test bearings) at the same *P_s_*. The predicted bearing flow rates are in good agreement with the test results.

**Figure 13:**
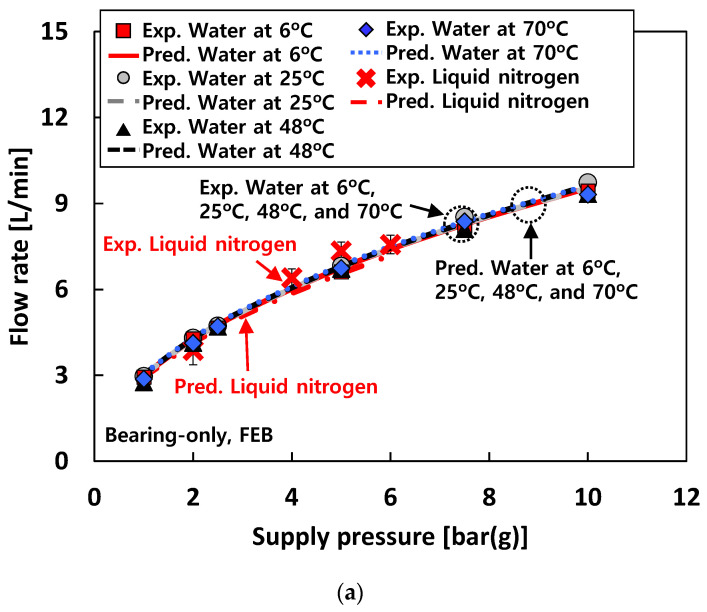

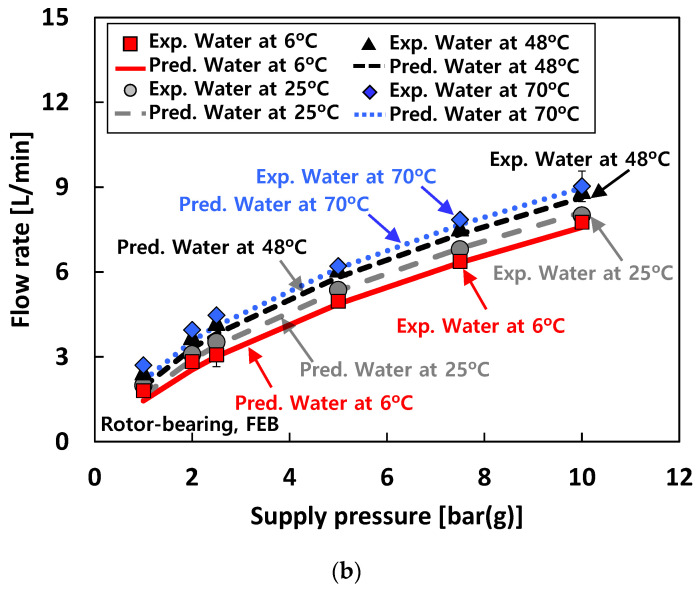
Predictions versus measurements. Test cases #2 and #3: bearing flow rate of free end bearing (FEB). (**a**) Bearing-only condition. (**b**) Rotor-bearing condition.

For test cases #2 and #3 (i.e., tests with water and liquid nitrogen, respectively), Figure 13a,b compare the predicted and measured flow rate of the free end bearing for the bearing-only (i.e., without rotor in the test bearings) and the rotor-bearing (i.e., rotor inserted in the test bearings) conditions, respectively. For the bearing-only condition, the water temperatures render nearly no effect on the bearing flow rates. Note that, for the bearing-only condition, the bearing flow rates for test case #3 (i.e., tests with liquid nitrogen) are quite similar to those for test case #2 (i.e., tests with water). On the other hand, for the rotor-bearing condition, the water temperature affects the bearing flow rate for test case #2. That is, the bearing flow rates increase with the water temperature. Predicted bearing flow rates for both the bearing-only and the rotor-bearing conditions are in good agreement with measurements.

**Figure 14:**
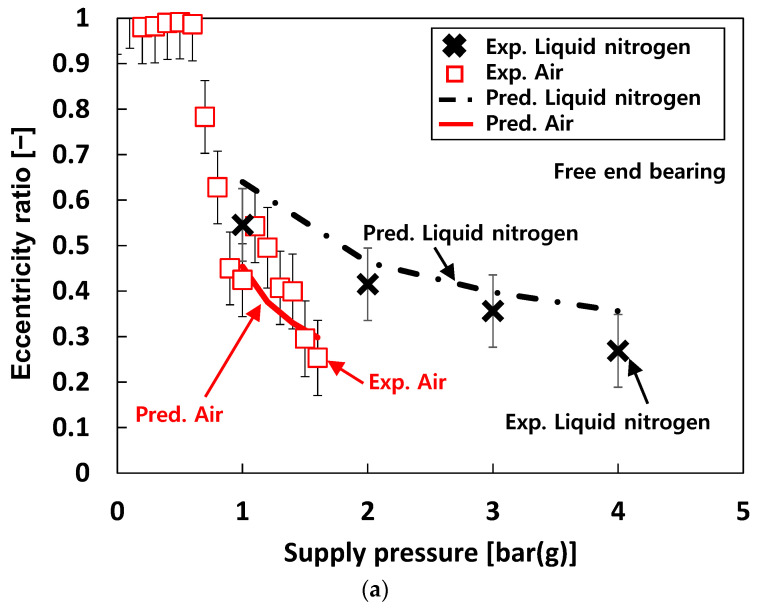

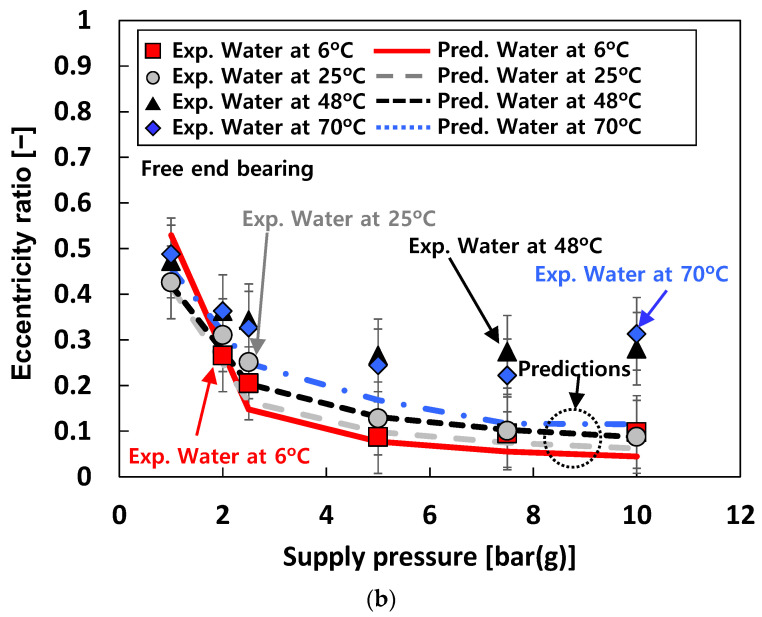
Predictions versus measurements. Test cases #1, #2, and #3: eccentricity ratio of free end bearing. (**a**) Test cases #1 and #3. (**b**) Test case #2.

Figure 14 compares the predicted and the measured eccentricity ratios of the free end bearing for test cases #1 through #3. The predicted eccentricity ratios noticeably decrease with *P_s_*. For test case #1 (i.e., tests with air), the predicted eccentricity ratios agree well with the test data. For test case #2 (i.e., tests with water), correlations between measurements and predictions become less favorable as the water temperature increases while those for 6 °C water are remarkable. For test case #3 (i.e., tests with liquid nitrogen), the predictions show a good agreement with the measurements even though the predicted eccentricity ratios are slightly larger than the test data.

**Figure 15:**
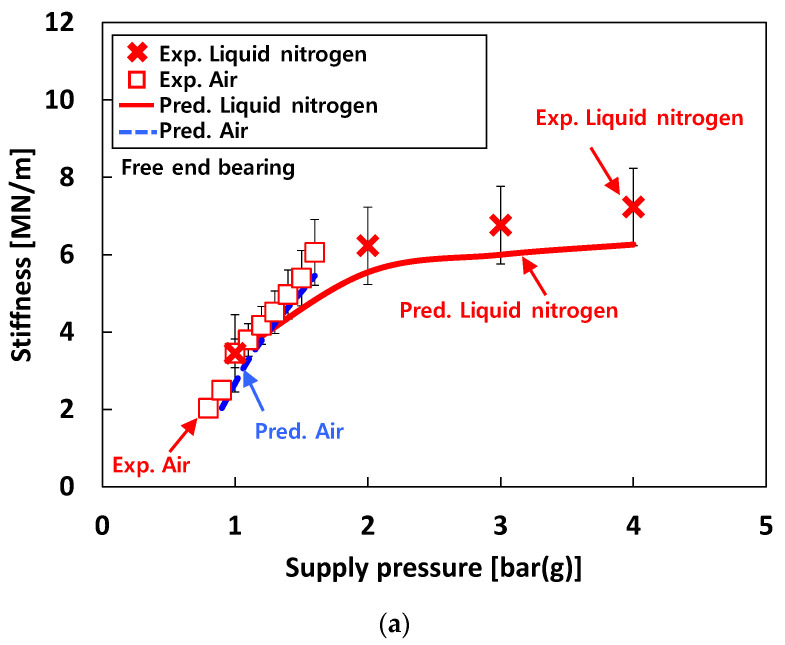

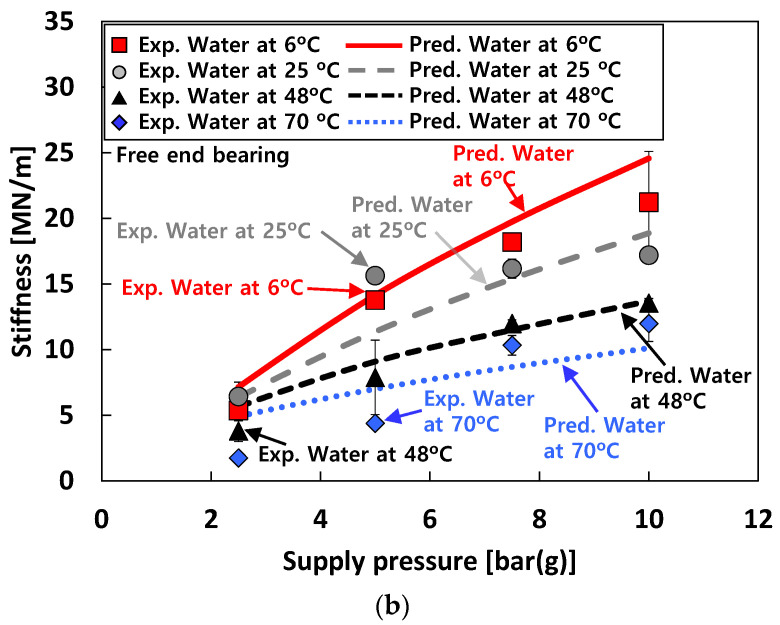
Predictions versus measurements. Test cases #1, #2, and #3: stiffnesses of free end bearing. (**a**) Test cases #1 and #3. (**b**) Test case #2.

Figure 15 compares the predicted and the measured bearing stiffnesses of the free end bearing for test cases #1 through #3. For test case #1 (i.e., tests with air), the predicted and measured stiffnesses increase linearly with *P_s_*, and the agreement between predictions and measurements is remarkable. For test case 2 (i.e., tests with water), in general, the predicted stiffnesses agree reasonably with the measurements. For test case #3 (i.e., tests with liquid nitrogen), the trends between measurements and predictions appear quite similar as *P_s_* increases. However, the comparisons between predicted and measured stiffnesses for test case #3 are less favorable than those for test cases #1 and #2. As discussed in the previous chapter (i.e., 4. Experimental Results), this is mainly due to the phase transition of liquid nitrogen from all-liquid to two-phase flow in the test bearings. 

## 6. Conclusions

The current work performs extensive tests to measure the static load characteristics of hydrostatic bearings, such as bearing flow rate, eccentricity ratio, torque, and stiffness, using various test fluids (i.e., air, water, and liquid nitrogen) at a non-rotating condition for increasing supply pressure (*P_s_*) into the test bearings. In addition, measurements are compared to predictions for validation of the bearing prediction model. The test results show that the static load characteristics of the test hydrostatic bearing strongly rely on test fluids, as well as their bearing inlet temperatures, and static load conditions. The measured bearing flow rates for the tests with air are much larger than those for the tests with water and liquid nitrogen. For the tests with water fed into the bearings, the measured bearing flow rates increase as the water temperature increases when the rotor is installed within the bearings (i.e., the rotor-bearing condition). However, when the rotor is removed from the test rig and measurements are conducted only with the bearings (i.e., the bearing-only condition), the measured bearing flow rates do not notably change with water temperature. Interestingly, the measured bearing flow rates for the tests with water are not quite different from those for the tests with liquid nitrogen. When water is used as a test fluid, as the water gets warmer, the measured bearing eccentricity ratios increase. The measured bearing eccentricity ratios for the tests with liquid nitrogen are higher than those for the tests with water. Bearing stiffnesses are identified by the rap test, as well as the excited vibration frequencies by pneumatic hammer instability for the tests with air and liquid nitrogen. The measured bearing stiffnesses for the tests with water gradually increase with fluid supply pressure into the bearings and decreases with the water temperatures. Bearing performance predictions are benchmarked against the comprehensive measurement data tested with air, water, and liquid nitrogen. The predicted bearing flow rate, eccentricity ratio, and stiffness are in notable agreement with the test data for various supply pressure conditions. Note that for the tests with liquid nitrogen, the comparisons between predictions and measurements clearly infer a phase transition of liquid nitrogen in the test bearings due to a large thermal gradient from the outside of the bearing housing to the thin film of the test bearings and relatively low fluid (i.e., liquid nitrogen) supply pressure into the test bearings. This evidence a need for employing a thermo-hydrodynamic model considering a two-phase fluid condition for more improved and accurate bearing characteristics predictions. The present work provides an extensive database on the static load characteristics of hydrostatic bearings lubricated with compressible (air), incompressible (water), and cryogenic (liquid nitrogen) fluids. Currently, comprehensive rotordynamic testing is underway to measure shaft motions of the present test rig for various fluid supply conditions while increasing rotor speed.

## Figures and Tables

**Table 1:** Measured dimensions and physical properties of test rotor and test bearing.

**Test Rotor**	**Value**
Material	SUS630
Outer diameter at bearing locations	59.896 (±0.002) mm
Length	590 mm
Mass center from rotor free end	293 mm
Polar moment of inertia (*I_p_*)	4.61 × 10^−3^ kg·m^2^
Transverse moment of inertia (*I_t_*)	2.12 × 10^−1^ kg·m^2^
Mass	10.40 kg
**Test Bearings**	**Value**
Material	SUS630
Outer diameter	100.000 (±0.002) mm
Inner diameter	60.000 (±0.002) mm
Axial length	25.00 (±0.002) mm
Radial clearance	0.052 (±0.002) mm
Orifice diameter	0.82 (±0.005) mm
Number of recesses	9
Axial and circumferential lengths of recess	9.86 mm (±0.003)

**Table 2:** Test cases (test fluids, fluid temperature, and measured/estimated parameters).

Test Case #	Test Fluid	Controlled Bearing Inlet Fluid Temperature, *T_s_* [°C]	Measured or Estimated Parameters
1	Air	25	Supply pressure, flow rate, orifice discharge coefficient, rotor centerline motion, torque, and stiffness
2	Water	6, 25, 48, and 70	Supply pressure, flow rate, orifice discharge coefficient, rotor centerline motion, torque, and stiffness
3	Liquid nitrogen	−197	Supply pressure, flow rate, orifice discharge coefficient, rotor displacement, and stiffness

## Data Availability

Not applicable.

## Abbreviations

**Nomenclature**	
*A_o_*	Orifice area (m^2^)
*C_d_*	Orifice discharge coefficients (-)
*C*	Bearing radial clearance (m)
*D*	Bearing diameter (m)
*D_rotor_*	Rotor diameter (m)
*d_orifice_*	Orifice diameter (m)
*f*	Frequency (Hz)
g	Flow function (-)
*G_x_, G_z_*	Turbulence parameters (-)
*h*	Film thickness at recess edge (m)
*h_recess_*	Recess depth (m)
*I_p_*	Polar moment of inertia (kg-m^2^)
*I_t_*	Transverse moment of inertia (kg-m^2^)
*k_1_*	Bearing stiffness of free end bearing (N/m)
*k_2_*	Bearing stiffness of drive end bearing (N/m)
*K*	Bearing stiffness
*L*	Bearing length (m)
*l*	Recess length (m)
*m_1_*	Mass acting on free end bearing (kg)
*m_2_*	Mass acting on drive end bearing (kg)
*M*	Rotor mass (kg)
*P*	Fluid film pressure (Pa)
*P_s_*	Supply pressure (Pa)
*P_r_*	Recess pressure (Pa)
*Q*	Flow rate (kg/s)
ℜ	Gas constant
*T_s_*	Supply fluid temperature (°C)
*U*	Rotor surface speed (m/s^2^)
*X, Y, Z*	Inertial coordinate system (m)
*κ*	Specific heat ratio of air (-)
*ρ*	Density (kg/m^3^)
*µ*	Viscosity (Pa-s)
*υ*	Normal fluid velocity to recess edge (m/s)
*Φ*	Flow equation
*ω*	Measured natural frequency (rad/s)
**Acronyms**	
FEB	Free end bearing
DEB	Drive end bearing
Exp.	Experiment
Pred.	Prediction
